# Supplementary material for: Fallopia japonica Root Extract Ameliorates Ovalbumin-Induced Airway Inflammation in a CARAS Mouse Model by Modulating the IL-33/TSLP/NF-κB Signaling Pathway
Source: Int J Mol Sci. 2023 Aug 7;24(15):12514. doi: 10.3390/ijms241512514 (PMC10420321; doi:10.3390/ijms241512514)
Supplement: Supplementary file 1 [file ijms-24-12514-s001.zip › ijms-2526408-supplementary.pdf]

Supplementary material for

Fallopia japonica Root Extract Ameliorates Ovalbumin-Induced Airway Inflammation in a CARAS Mouse Model by Modulating the IL-33/TSLP/NF-κB Signaling Pathway

Juan Jin [1], Yan Jing Fan [1,†], Thi Van Nguyen [1], Zhen Nan Yu [1], Chang Ho Song [1,2], So-Yong Lee [3,4], Hee Soon Shin [3,4], Ok Hee Chai [1,2,*]

[1] Department of Anatomy, Jeonbuk National University Medical School, Jeonju, Jeonbuk, 54896, Republic of Korea
[2] Institute for Medical Sciences, Jeonbuk National University Medical School.Jeonju,54896, Jeonbuk, Korea.
[3] Department of Food Biotechnology, Korea University of Science and Technology, Daejeon 34113, Korea
[4] Department of Food Functionality Research, Korea Food Research Institute, Wanju 55365, Korea.
* Correspondence: okchai1004@jbnu.ac.kr (O.H.Chai); Tel.: +82-6-3270-3109
† Current address: School of Medicine, Liaocheng University, Liaocheng 252000, China.

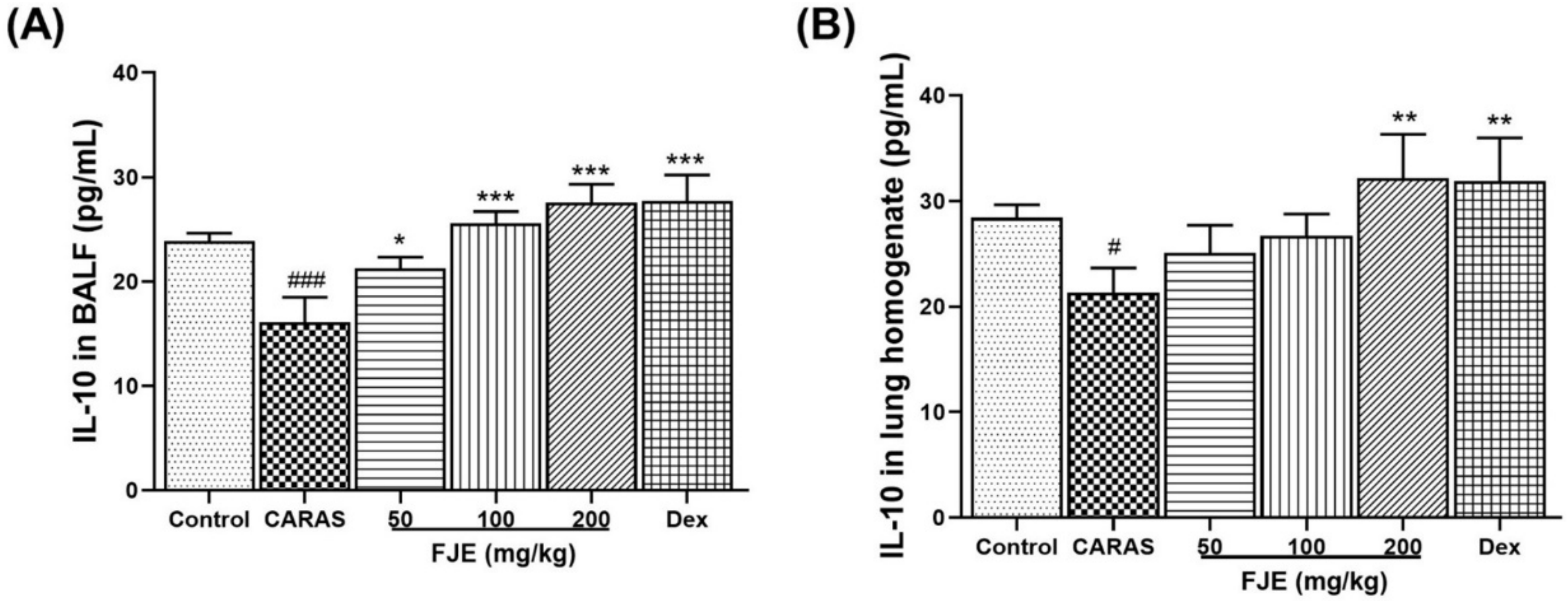

Figure S1. Influence of FJE on IL-10 in the BALF and lung homogenate. Mice were administered saline, Dex (1.5 mg/kg), or FJE (50, 100, 200 mg/kg) once a day for 16 days. Data represent mean ± SEM (n=6/group). #$p < 0.05$, ###$p < 0.001$ compared with control, *$p < 0.05$, **$p < 0.01$, ***$p < 0.001$ vs. CARAS. Dex, Dexamethasone.

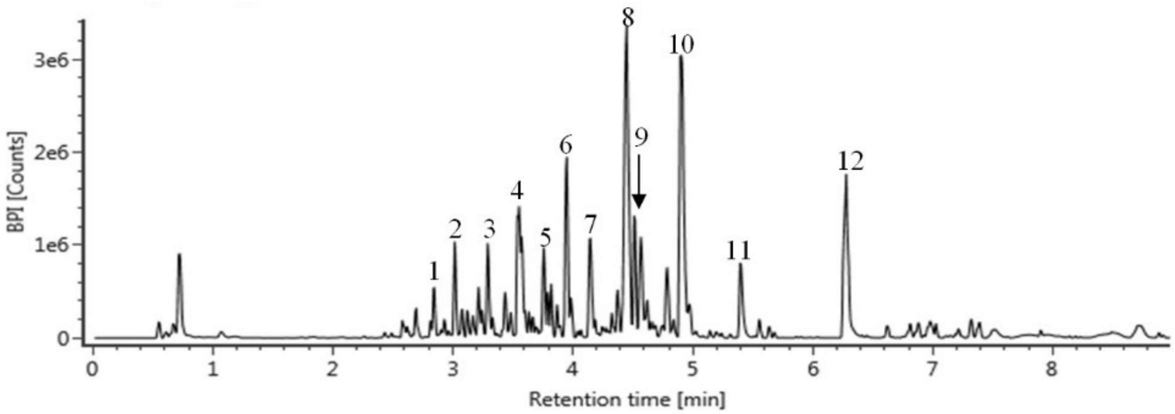

No.	RT	Compounds	Obserbed mass [M-H]	Mass error	Mass fragment
1	2.85	Procyanidin B	577.1343	-0.9	425, 407, 287
2	3.02	Cianidanol / epicatechin / catechin	289.0718	0	137, 125
3	3.3	procyanidin B-gallate	729.145	-1.1	441, 407, 289
4	3.55	Piceid	389.1245	0.3	227
5	3.76	resveratrol 3-(6' '-galloyl)-O-β-D-glucopyranoside	541.135	-0.1	313, 227, 169
6	3.95	emodin-glucoside 1	431.0988	0.4	269
7	4.15	Vitexin sulfate / isovitexin sulfate	511.0553	0.1	413, 269
8	4.45	emodin-glucoside 2	431.0989	0.5	269
9	4.57	pelargonidin 3-glucoside malonate	517.0984	-0.3	473, 269
10	4.91	marmesin / decursinol	245.0817	-0.3	230, 159
11	5.4	sulfemodin-glucoside	349.0024	0	269
12	6.28	emodin	269.0455	0	241, 225

Figure S2. Representative mass chromatograms of *Fallopia japonica* root extract (FJE). The mass chromatograms were obtained using UPLC-Q-TOF-MS with negative mode, a total of 12 compounds were identified. Among these resveratrol, emodin-glucoside 1, emodin glucoside 2, procyanidin, and other polyphenols potentially suppress airway inflammation.
